# Infectious Salmon Anemia and Farm-Level Culling Strategies

**DOI:** 10.3389/fvets.2019.00481

**Published:** 2020-01-15

**Authors:** Lars Qviller, Anja B. Kristoffersen, Trude M. Lyngstad, Atle Lillehaug

**Affiliations:** Norwegian Veterinary Institute, Oslo, Norway

**Keywords:** infectious salmon anemia (ISA), salmon aquaculture, biosecurity, culling, disease transmission

## Abstract

Infectious salmon anemia (ISA) is an infectious disease, and outbreaks must be handled to avoid spread between salmon sea farms. Intensive culling at infected farms is an important biosecurity measure to avoid further spread but is also a costly intervention that farmers try to avoid. A lack of action, however, may lead to new outbreaks in nearby salmon sea farms, with severe impacts on both economy and animal welfare. Here, we aim to explore how a time delay between a detected outbreak and the culling of both infected cages and entire farms affects the further spread of the disease. We use a previously published model to calculate how many salmon sea farms were directly infected in each outbreak. To investigate the effect of culling on the further spread of disease, we use the number of months elapsed from the detected outbreak to (a) the first cage being depopulated, and (b) to the entire salmon sea farm being depopulated as predictors of how many new farms the virus was transmitted to, after controlling for contact between the farms. We show that the lapse in time before the first cage is depopulated correlates positively with how many new salmon sea farms are infected, indicating that infected cages should be culled with as little time delay as possible. The model does not have sufficient power to separate between culling of only cages assumed to be infected and the entire farm, and, consequently, provides no direct empirical evidence for the latter. Lack of evidence is not evidence, however, and we argue that a high probability of spread between cages in infected salmon sea farms still supports the depopulation of entire farms as the safest option.

## 1. Introduction

The production of farmed Atlantic salmon is a growing industry that has developed into a large international business over the last few decades. Norway is currently the largest actor in this industry, with an annual production of about 1.2 million metric tons and with somewhere between 350 and 450 million salmon in marine farms along the Norwegian coast at any time during the last 4 years (1). An average Norwegian salmon sea farm is stocked with almost one million smolt (calculated using data gathered from https://www.fiskeridir.no). The continuously increasing volumes have led to concerns about environmental and pathogenic impacts (1, 2). The current political aim of a quintupling of the aquaculture production by 2050 (2, 3) will lead to an increase in fish populations susceptible to pathogenic disease agents, and more efficient multiplication and dissemination of such agents may be a consequence. It is therefore of great importance to develop effective disease control measures and to evaluate their effect.

Marine culture of salmonids usually takes place in production units consisting of between one and 15 cages in proximity to each other. The cages may be constructed as open net pens outlined by steel squares that are welded together or by individual plastic rings, typically with a circumference of 160 m. The cages are held in a limited area, managed together by a central fleet with workers, equipment, and a shared system for fodder distribution. Such production units are commonly referred to as salmon sea farms. Each such farm must be approved by the authorities and registered in the Norwegian aquaculture register.

Infectious salmon anemia (ISA) is a serious viral disease in farmed Atlantic salmon, and it may also infect rainbow trout. It is caused by virulent HPR-deleted strains of the ISA virus (4), possibly evolving from non-virulent strains of the ISA virus, which is called ISAV-HPR0 (5, 6). The disease is listed as notifiable by the World Animal Health Organization (OIE, 4). An outbreak of ISA develops slowly, but the majority of the fish in an infected population may succumb during the production cycle. Cumulative mortalities as high as 90% have been reported in farms (4, 7–9). The disease is contagious, and while there is an ongoing debate over whether it can transmit vertically (4, 9, 10), its ability to spread horizontally is well established (4, 9, 11, 12). The disease can spread to other salmon sea farms, using pathways such as passive transmission in the water or with contaminated equipment, boat traffic, or the movement of fish (12–18).

The disease has even shown its potential to destroy entire salmon farming industries, tragically exemplified with the epidemics in Chile in 2007–2009 (18, 19) and in the Faeroe Islands in 2000–2005 (20). The Norwegian aquaculture industry was the first to experience challenges with ISA, with the first outbreak described in 1984 (8). As many as 80 new infected farms were reported in 1990, at the peak of historical outbreaks in Norway (21). The ISA problem in Norway in the nineties was mitigated through regulations including compulsory fish health controls, disinfection in hatcheries, regulation of live fish transportation, “all-in-all-out” (only one generation of fish at the same time in each salmon sea farm), and following between generations (9). Due to its status as a listed disease, outbreaks of ISA call for mandatory disease control measures to be taken by the Norwegian Food Safety Authority. These measures include establishing a disease control zone with surveillance of fish populations, culling of infected cages or entire farm populations, and ensuring a period of coordinated fallowing of the entire zone after depopulation (4, 22).

Although the contemporary issue with ISA outbreaks in Norway is much less severe than in the nineties, the industry still faces between zero and seven outbreaks (~ 0.7% of each generation) with unknown source of infection, every year on average (23). These initial outbreaks may cause local epidemics of secondary outbreaks. Aldrin et al. (12) have developed a transmission model that uses factors such as geographic distances between salmon sea farms, genetic similarities between virus isolates, and the number of fish in both the susceptible and infected salmon sea farms. This model can be used to describe local epidemics in order to substantiate whether the outbreak in one farm has spread to another. Note that this model (or any model derived from it) did not include the effects of water currents. The model infers infectious contact over 6 months prior to the outbreak, and we assume that hydrodynamic patterns even out over the period. Further, the mechanisms behind transmission of ISA are not known in enough detail to include spatiotemporal movements of the water.

There is evidence suggesting that all cages in a salmon sea farm with ISA are highly likely to contract the disease if one cage is infected and that the functional epidemiologic unit of an ISA outbreak is the entire salmon sea farm and not the single cage (19).

Due to the slow development of the disease, the individual salmon sea farm may occasionally benefit if production is continued, despite the increased mortality. It is therefore not surprising that some farms want to delay the culling of the population, sometimes by almost a year, in order for the fish to reach harvesting size and thereby reduce economic losses. However, if neighboring salmon sea farms are infected, the total losses may increase correspondingly, and if the disease is not managed, the situation may escalate. A key element in preventing the disease from further spread is the early removal of infected populations, but the economic cost of such actions often leads to culling only of cages with clinical signs and/or confirmed diagnosis (18, 24). Such a practice is highly questionable, as fish are known to be infectious several weeks or even months before displaying any clinical signs (9, 12, 15).

In the present paper, we use information from ISA epidemics to explore how the time elapsed before the culling of only cages with a confirmed ISA diagnosis and before the culling of entire salmon sea farms affects the probability of new secondary outbreaks in neighboring farms. Note that we did not include the time from infection to when the outbreak was suspected because precautionary measures like culling cannot be affected without a confirmed diagnosis. ISA detection and monitoring are outside the scope of this paper.

More specifically, we intend to shed light on the following two hypotheses:

- **Hypothesis 1:** The risk of secondary cases will increase with the length of time that salmon sea farms hold fish diagnosed with ISA.
- **Hypothesis 2:** Culling of the entire salmon sea farm is a better precautionary measure than only culling cages with high mortality or a confirmed diagnosis.

## 2. Methods

### 2.1. Cases of Infectious Salmon Anemia

The Norwegian Veterinary Institute is the national reference laboratory for fish diseases and is also an OIE reference laboratory for infectious salmon anemia (ISA). The diagnosis of ISA should be confirmed by the Norwegian Veterinary Institute, who report all cases to the Norwegian Food Safety Authority (22). Fish health personnel are mandated to report suspicion of ISA to the Norwegian Food Safety Authority. Suspicion may be due to clinical signs or increased mortality in fish. Samples from farms with suspected ISA must be sent to the Norwegian Veterinary Institute for identification of the ISA virus, including DNA analyses for genetic characterization. The outbreak remains a suspected case until the Norwegian Veterinary Institute confirms the diagnosis of ISA. The Norwegian Food Safety Authority will then declare the farm infected and enforce disease control measures, including forced culling, according to governmental regulations on the prevention and control of infectious diseases in aquatic animals (22). We use confirmed ISA outbreaks as cases of ISA in the present study, and we take the date when suspicion of ISA was reported in cases that were later confirmed as the date of detection of ISA.

### 2.2. Farm Production Data

All Norwegian salmon sea farms must report key production statistics every month to the Directorate of Fisheries. These data are to be reported both on the farm level and for individual cages in the farm. The data include information about salmon biomass and number of fish, mortality, and when the salmon sea farm was stocked with fish. The start weight when the fish were stocked is also included. We defined a cohort as relocated if the fish had a start weight larger than 250 g, implying that they originated from other salmon sea farms and that they may have been re-classified from other cohorts (12, 25). We have used the number of fish to establish the culling practices in each outbreak, namely how many months it took from suspicion until the infected farm no longer held fish and how many months it took before the first cage was empty. The cages were not always reported with unique identifiers, leaving specific cage identities impossible to follow in many cases. We have therefore not been able to collect trustworthy data on when all cages with confirmed diagnosis or high mortality (i.e., infected cages) were culled. To overcome this issue, we assumed that if the farmers took action against the outbreak, they would depopulate the infected cages first. Under this assumption, the time elapsed before the first cage was emptied would correlate strongly with how quickly they depopulated all fish from cages with high mortality or confirmed diagnosis. We therefore used the time elapsed before the first cage was emptied as a proxy for depopulation of the infected cage/cages. We used the salmon abundance data, when they stocked the farms, whether they relocated fish, as well as seaway distances between farms to calculate the transmission impact of an outbreak to the surrounding area. The transmission impact is defined as the outbreak's *area susceptibility*. In addition to this, we used genetic similarities between the outbreak's virus strains and the date a new salmon sea farm was reported to be under suspicion of ISA to establish how many farms were directly infected in each outbreak.

### 2.3. The Transmission Model

Here, we have used all cases of ISA with a sequenced genome in salmon sea farms that have been confirmed by the Norwegian Food Safety Authority as our study population. The chronology, production statistics, seaway distances, and genetic similarities between virus isolates in all infected salmon sea farms were used to establish transmission pathways according to Aldrin et al. (12) (Equation 1). A seaway distance between two farms is the shortest distance between farms in the sea, around landmasses and islands, calculated using the gdistance package in R (26). The transmission model identified the cases that were most likely primary outbreaks with an unknown source of infection and detailed pathways through secondary outbreaks from this ultimate infection source. These transmission networks hold information on where a secondary outbreak was likely to have its direct source, and, consequently, how many new salmon sea farms were directly infected in each outbreak. This is key information for the present study, and the number of salmon sea farms infected from each farm-level outbreak (secondary outbreaks) was used as the response variable in all of the statistical analyses herein. Note that we did not include outbreaks confirmed after July 2017 in the response to avoid outbreaks that may lead to new epidemics that are not yet fully resolved. We did, however, include all confirmed outbreaks in 2017 when we calculated the number of secondary outbreaks. The data used herein includes all confirmed ISA cases from January 1, 2004 to July 31, 2017. The list of confirmed ISA cases we used here is the same as was used in a recently published paper (23).

We use the previously published transmission model to establish the most likely transmission pathways. Aldrin et al. (12) used a function to establish the rate of infection in any salmon sea farm *i* of a given virus genotype *g* in a specific month *t*, termed λ_*gi*_(*t*). The function is based on the factors described under production statistics and ISA data, in addition to genetic similarities between isolated virus genotypes from the infected salmon sea farms. Genetic similarities were calculated using the Kimura 2 parameter model for nucleotide substitution, using the 5 part of the HE gene. We use the following formula to calculate the rate of infection in salmon sea farms from Aldrin et al. (12):

(1) $$\begin{array}{r} \lambda_{gi}\left(t\right)=S_{i}\left(t\right)\cdot \lambda_{b}\left(t\right)\cdot \lambda_{ix}\left(t\right)\cdot \left[\lambda_{gi}^{d}\left(t\right)+\lambda_{gi}^{o}\left(t\right)\right] \end{array}$$

where *S*_*i*_(*t*) denotes whether a salmon sea farm *i* is susceptible (1) or not (0), λ_*b*_(*t*) denotes the baseline rate, a time-varying rate of infection common for all salmon sea farms and independent of space. This factor was added to the models because ISA may appear in salmon sea farms with no known source of infection (23). λ_*ix*_(*t*) is a factor proportional to the susceptibility of salmon sea farm *i* and is functionally related to fish abundance and the characteristics of the fish cohort at salmon sea farm *i* at time *t*. $\lambda_{gi}^{d}\left(t\right)$ is the relative rate of infection at time *t* from infectious salmon sea farms in the neighborhood and is related to the seaway distances to infectious farms and the genetic similarity between the given genotype *g* and the genotypes of ISA viruses isolated from the infectious farms. $\lambda_{gi}^{o}\left(t\right)$ describes the relative rate of infection through non-specified pathways.

Equation (1) can be expanded to equation 3.6 from Aldrin et al. (12):

(2) $$\begin{array}{l} \;\lambda_{gi}(t)=S_{i}(t)\;\cdot \;\lambda_{b}(t)\;\cdot \;{\left(\beta^{a}\right)}^{x_{i}^{a}(t)}\;\cdot \;{\left(\beta^{m}\right)}^{x_{i}^{m}(t)}\;\cdot \;{\left(\beta^{r}\right)}^{x_{i}^{r}(t)}\;\cdot \;{\left(x_{i}^{n}(t))\right)}^{\beta^{n}}\\ \times \;\left[\sum_{j\neq i}\{exp(-\phi \;\cdot \;d_{ij}^{s})\;\cdot \;exp(-\omega \;\cdot \;d_{ij}^{g})\;\cdot \;{\left(z_{j}^{n}(t)\right)}^{\alpha^{n}(t)}\;\cdot \;I_{j}(t)\}+\theta \right] \end{array}$$

Here, we see that λ_*ix*_(*t*) is expanded into the specific properties ${\left(\beta^{a}\right)}^{x_{i}^{a}(t)}\;\cdot {\left(\beta^{m}\right)}^{x_{i}^{m}(t)}\;\cdot {\left(\beta^{r}\right)}^{x_{i}^{r}(t)}\;\cdot {\left(x_{i}^{n}(t)\right)}^{\beta^{n}}$, which are further explained in Equation (5). The contribution from the surrounding salmon sea farms *j* is expanded into:

- a spatial component $exp\left(-\phi \cdot d_{ij}^{s}\right)$ and the infectiousness of the salmon sea farm ${\left(z_{j}^{n}\left(t\right)\right)}^{\alpha^{n}\left(t\right)}$, which are both explained in equation 6,
- the genetic component $exp\left(-\omega \cdot d_{ij}^{g}\right)$, where $d_{ij}^{g}$ is the genetic distance between virus isolates from salmon sea farm *j* and salmon sea farm *i* and ω is a parameter that expresses the effect of the genetic distance on the risk of infection,
- *I*_*j*_(*t*) indicates whether the salmon sea farm was infected or not (1 or 0), and
- non-specified pathways $\lambda_{gi}^{o}\left(t\right)$ are replaced by the constant θ.

We define salmon sea farm *j* that contributes the most to $\lambda_{gi}^{d}\left(t\right)$ as the most likely source of outbreak *i*. Please refer to Aldrin et al. (12) for a more detailed description of the transmission model and parameter estimation procedures.

### 2.4. Area Susceptibility

Every outbreak was situated in a unique situation in space and time, with a varying density of surrounding salmon sea farms. Some of the surrounding salmon sea farms may have been susceptible to infection, while others were already infected. The probability of further spread from an outbreak is therefore not only defined by the time delay before culling but also by the density and proximity of surrounding salmon sea farms. In addition, the distance between each source outbreak and the surrounding susceptible farms, as well as the individual properties of both the infectious and the susceptible farms, may have affected the rate of transmission from this source outbreak. Based on the factors mentioned above, we have calculated the contact with the surrounding salmon sea farms, which we term the *area susceptibility*.

Thus, the *area susceptibility* for an ISA outbreak related to a specific infected salmon sea farm in a given month is a continuous function of the following variables: number of fish in the surrounding salmon sea farms, distance to the surrounding salmon sea farms that are not already infected, number of fish in the infected farm, stocking season, and whether fish in the susceptible cohort have been relocated. Here, we define the relative susceptibility of an uninfected salmon sea farm to a source outbreak as the spatial relationship given by the transmission model. We used this relationship as a measure of connectedness between a source outbreak and a susceptible salmon sea farm, regardless of whether it became infected or not at a later point in time. We calculated the area susceptibility for the month prior to suspicion of ISA to avoid culling practices after suspicion affecting the calculations.

Our calculation of area susceptibility relies heavily on the calculations from Aldrin et al. (12), but note that Aldrin et al. (12) investigated the rate of infection of a susceptible salmon sea farm from all surrounding infected salmon sea farms. The area susceptibility, on the other hand, is the spatial component of the rate of infection *from* a salmon sea farm to all surrounding susceptible salmon sea farms. We must therefore recalculate the equation to serve this purpose.

We get the specific transmission rate $\lambda_{ij}^{spec}\left(t\right)$ between any salmon sea farm *i* and an outbreak *j* in a given month *t* by a reformulation of formula 3.1 in Aldrin et al. (12):

(3) $$\begin{array}{r} \lambda_{ij}^{spec}\left(t\right)=S_{i}\left(t\right)\cdot \lambda_{ix}\left(t\right)\cdot \lambda_{xj}^{d}\left(t\right) \end{array}$$

Here,

- *S*_*i*_(*t*) is the risk indicator, a binary variable that takes the value 0 if the salmon sea farm is empty or already infected, and 1 if it is susceptible.
- λ_*ix*_(*t*) is a factor proportional to the susceptibility of salmon sea farm *i* and is and functionally related to fish abundance and the characteristics of the fish cohort at salmon sea farm *i* at time *t*. A susceptible farm is a salmon sea farm without a known ISA outbreak that holds fish.
- $\lambda_{xj}^{d}\left(t\right)$ is a parameter that describes the infectiousness of salmon sea farm *j*, penalized by the seaway distance between the infected farm *j* and the susceptible farm *i*.

Note that this approach has discarded the genetic similarity component, the baseline rate, and the rate of infection through other pathways from the formulation in Aldrin et al. (12). These factors were not considered as part of the spatial relationship.

The area susceptibility in month *t* of an outbreak *j* is thus the sum of $\lambda_{ij}^{spec}\left(t\right)$ for all surrounding susceptible farms *i*. This relationship takes the form:

(4) $$\begin{array}{r} \lambda_{j}\left(t\right)=\underset{i}{\sum }\lambda_{ij}^{spec}\left(t\right)=\underset{i}{\sum }S_{i}\left(t\right)\cdot \lambda_{ix}\left(t\right)\cdot \lambda_{xj}^{d}\left(t\right) \end{array}$$

#### 2.4.1. Farm Susceptibility

Aldrin et al. (12) found that the susceptibility of a salmon sea farm depended on the salmon sea farm's fish abundance and whether the farm was stocked in autumn, spring, or a mix of the two or was relocated. The susceptibility was expressed by the formula:

(5) $$\begin{array}{r} \lambda_{ix}\left(t\right)={\left(\beta^{a}\right)}^{x_{i}^{a}\left(t\right)}\cdot {\left(\beta^{m}\right)}^{x_{i}^{m}\left(t\right)}\cdot {\left(\beta^{r}\right)}^{x_{i}^{r}\left(t\right)}\cdot {\left(x_{i}^{n}\left(t\right)\right)}^{\beta^{n}} \end{array}$$

In our study, the susceptibility of a salmon sea farm λ_*ix*_(*t*) depends on the binary variables indicating whether the cohort *i* was stocked in autumn $x_{i}^{a}\left(t\right)$, both autumn and spring (mixed) $x_{i}^{m}\left(t\right)$, spring (autumn and mixed variables are both set to 0), or whether the cohort was relocated (stocking season unknown) $x_{i}^{r}\left(t\right)$ and on the number of fish in the cohort in month *t*, denoted by $x_{i}^{n}\left(t\right)$. The β parameters express the effect of these parameters in log-linear relationships.

#### 2.4.2. Distances and Infectiousness of an Outbreak

Aldrin et al. (12) defined the rate of infection from a salmon sea farm $\lambda_{gi}^{d}\left(t\right)$, as a combination of distance between the infectious and susceptible salmon sea farms, the genetic similarities, and the infectiousness of the infected farm. Here, we have only considered the spatial component $\lambda_{xj}^{d}\left(t\right)$ of this factor, which is expressed by:

(6) $$\begin{array}{r} \lambda_{xj}^{d}\left(t\right)=exp\left(-\phi \cdot d_{ij}^{s}\right)\cdot {\left(z_{j}^{n}\left(t\right)\right)}^{\alpha^{n}} \end{array}$$

- $d_{ij}^{s}$ is the seaway distance between infectious salmon sea farm *j* and susceptible salmon sea farm *i*, while ϕ expresses the effect of the seaway distance on the risk of infection.
- ${\left(z_{j}^{n}\left(t\right)\right)}^{\alpha^{n}}$ represents the infectiousness of the infected salmon sea farm *j*, which depends on the fish abundance *z* of the farm at time *t*. The parameter α^*n*^ express the effect (α) of fish abundance (*n*) on infectiousness.

#### 2.4.3. Full Model for Area Susceptibility

The full model for area susceptibility in month *t*, λ_*ij*_(*t*) takes the form

(7) $$\begin{array}{r} \begin{array}{l} \lambda_{j}(t)=\underset{i\neq j}{\sum }[S_{i}(t)\cdot {\left(\beta^{a}\right)}^{x_{i}^{a}(t)}\cdot {\left(\beta^{m}\right)}^{x_{i}^{m}(t)}\cdot {\left(\beta^{r}\right)}^{x_{i}^{xr}(t)}\cdot {\left(x_{i}^{n}(t)\right)}^{\beta^{n}}\\ \;\cdot exp(-\phi \cdot d_{ij}^{s})\cdot {\left(z_{j}^{n}(t)\right)}^{\alpha^{n}}] \end{array} \end{array}$$

λ_*j*_(*t*) is the monthly rate of infection from the infected salmon sea farm *j* to all the susceptible farms *i* in the area in month *t*.

Here we base our calculations on the previously published paper that includes estimation of all parameters. All parameter estimates used in the area susceptibility model are presented in Table 1 in the present paper, and estimation methods are thoroughly explained in Aldrin et al. (12).

**Table 1:** Parameter estimates for the area susceptibility model [from Aldrin (12)].

**Effect of:**	**Symbol**	**Estimate**	**Lower CI**	**Upper CI**
Seaway distance	ϕ	0.095	0.046	0.145
Autumn cohort	β^*a*^	0.44	0.17	1.14
Mixed cohort	β^*m*^	1.12	0.52	2.39
Relocated cohort	β	1.30	0.65	2.62
Susc. cohort size	β^*n*^	0.57	0.11	1.02
Inf. cohort size	α^*n*^	2.71	0.13	5.55

### 2.5. Statistics

The response variable in all models was the number of secondary ISA outbreaks estimated to be transmitted directly from each source outbreak in our data set. The number of new outbreaks is an integer (count) variable. Two-thirds of the outbreaks did not lead to further infections, and the response seemed to be zero-inflated. We therefore performed model selection using the negative binomial distribution and the Poisson distribution, both with and without zero inflation. Zero inflation was implemented in its simplest form, with an intercept only.

**Figure 1:**
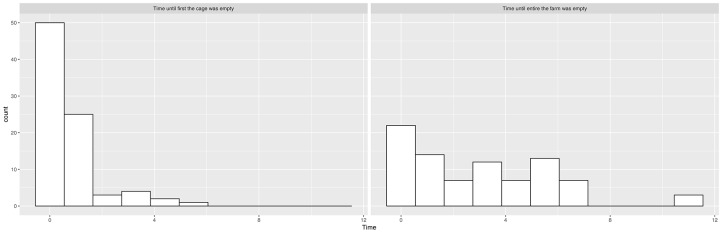
Histograms showing the distribution of time elapsed from suspected ISA outbreak until the first cage is empty and time elapsed from suspected ISA outbreak until the entire salmon sea farm is empty.

We see from the histograms in Figure 1 that salmon sea farms culling the first cage or the entire farm within the first month were overrepresented, and there is little continuous signal in these variables, especially for cages. We therefore transformed them into binary categories, indicating whether or not the first cage or the entire farm was culled within the first month. We also created a categorical variable called “culling” that indicated whether the culling of the entire farm was performed within the first month, whether the culling of the first cage (but not the rest of the farm) was performed within the first month, and whether the culling was further delayed.

Thus, we had six alternative explanatory variables before model selection: the five delay variables (1) the number of months from detected outbreak to the entire salmon sea farm being empty, (2) whether the entire salmon sea farm was empty after the first month (3) the number of months from detected outbreak until the first cage was empty, (4) whether the first cage was empty after the first month, (5) culling, and, finally, (6) the area susceptibility, with a log transformation to approximate normality. To avoid infinite negative numbers in cases where the area susceptibility was zero, we added 1 to the variable prior to log transformation. This was the case for one outbreak.

To understand which factors could explain the number of secondary outbreaks each outbreak would lead to, we performed a forward and a backward model selection procedure. The variable pairs 1, 2 and 3, 4 were alternative representations of the same variables, and only one of the variables in the pairs were included in the same model during model selection.

Interaction terms with area susceptibility were included as the final step of the model selection procedure. A positive interaction between a delay variable and area susceptibility would imply that the effect of contact with salmon sea farms in the proximity depended on how long the farm held fish that were potential viral shedders.

Model comparisons were performed using the Akaike information criterion (AIC), and the final models were compared using the Vuong test in the pscl-package in R (27). All analyses and the handling of data were performed using R version 3.4.1 (28), and the negative binomial regression analyses were performed using the R extension package MASS (29). Zero inflation was explored using the pscl-package in R (27). Sensitivity analysis and marginal effects with confidence intervals for the visualization of models were estimated using a non-parametric bootstrap procedure from the boot package in R (30, 31). The models were rerun and predicted 10,000 times on 85 samples with replacement from the original data (i.e., the original and sampled data frames were equal in size). Ninety five percentage percentile confidence estimates were retrieved using the boot.ci function in the boot package (31).

## 3. Results

Our data included 85 ISA outbreaks with traced epidemics between 2004 and 2017. Fifty-four outbreaks did not lead to known new infections, 20 source outbreaks infected one new salmon sea farm, while 11 source outbreaks spread to more than one new salmon sea farm, with a maximum of five secondary cases (Table 2). Note that the outbreaks spread between salmon sea farms, sometimes in small epidemics, as a chain reaction. Some outbreaks could therefore be secondary to one source outbreak but could still be the source of another.

**Table 2:** List of how many new outbreaks each ISA outbreak produced according to the transmission model in Aldrin et al. (12).

**Estimated further spread**	**Count**
0 further infections	54
1 further infection	20
2 further infections	5
3 further infections	2
4 further infections	3
5 further infections	1

Model selection revealed that the best model framework included the Poisson error distribution with zero inflation and that all competing models explained the number of secondary outbreaks as a response of area susceptibility and culling. The Vuong test showed no significant difference between the three best models; they were therefore interpreted as equally well fitted (Vuong test, AIC-corrected p: Top-ranked vs. alternative model 1 *p* = 0.35, top-ranked vs. alternative model 2 *p* = 0.29, alternative model 1 vs. alternative model 2. *p* = 0.39). Model parameters for the count part of the best and the two alternative models are shown in Table 3. All three models included a significant positive effect of area susceptibility, showing that infectious contact with surrounding farms increased the probability of transmission.

**Table 3:** The output (parameter estimates, standard errors, *z*- and *p*-values) of the count part of the top-ranked and the two alternative models.

	**Estimate**	**Std. Error**	***z* value**	**Pr(**>|*z*|**)**
**The top-ranked model** AIC: 176.9				
(Intercept)	−0.75	0.42	−1.77	0.08
Quick Cage Culling	−1.21	0.87	−1.40	0.16
Quick Farm Culling	0.51	0.56	0.91	0.36
Area Susceptibility (log)	1.84	0.53	3.49	0.00
Quick Cage Culling : Area Susceptibility (log)	−0.92	1.04	−0.88	0.38
Quick Farm Culling : Area Susceptibility (log)	−1.62	0.69	−2.33	0.02
**Alternative model 1** AIC: 178.1				
(Intercept)	−0.06	0.30	−0.19	0.85
Quick Cage Culling	−1.76	0.51	−3.48	0.00
Quick Farm Culling	−0.51	0.36	−1.41	0.16
Area Susceptibility (log)	0.79	0.33	2.42	0.02
**Alternative model 2** AIC: 179.5				
(Intercept)	−0.71	0.43	−1.66	0.10
Quick Culling	−0.10	0.53	−0.20	0.84
Area Susceptibility (log)	1.81	0.53	3.41	0.00
Quick Cage Culling : Area Susceptibility (log)	−1.43	0.68	−2.11	0.04

**Figure 2:**
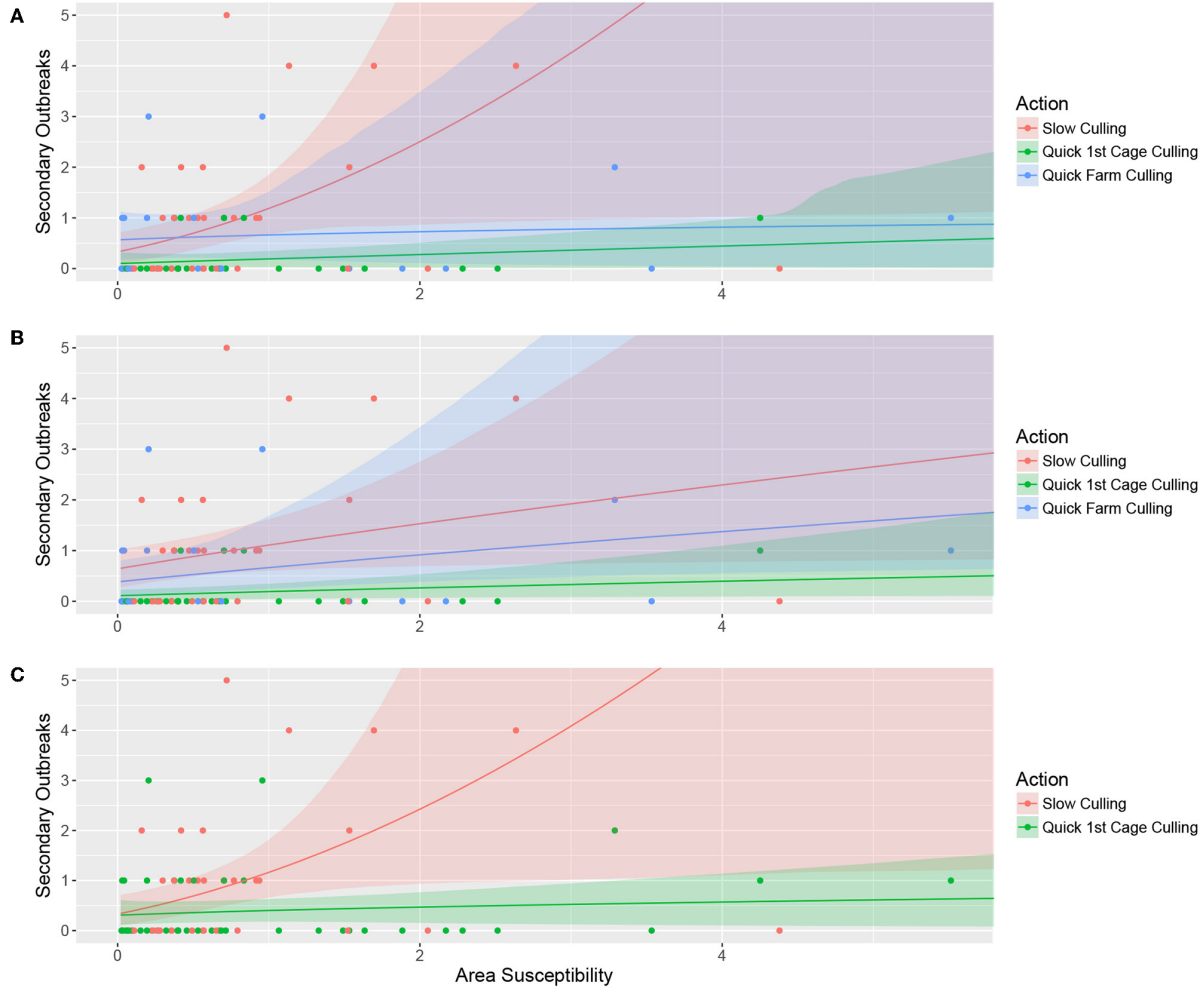
Bootstrapped model predictions (10,000 permutations) for the top-ranked model **(A)**, alternative model 1 **(B)**, and alternative model 2 **(C)**. Panel A illustrates the model where the number of secondary outbreaks is explained by the three-category variable “culling” in interaction with contact with other salmon sea farms (Area susceptibility), **(B)** illustrates the number of secondary outbreaks explained by the three-category variable “culling” and contact with other salmon sea farms without interaction, and **(C)** shows the number of secondary outbreaks explained by the two-category variable representing quick cage culling or not in interaction with contact with other salmon sea farms (Area susceptibility). The color-shaded areas illustrate the bootstrapped confidence intervals for the respective prediction line, and the points illustrate the raw data.

*The response variable in all models is the number of secondary outbreaks each outbreak led to. The response variables are contact with other salmon sea farms in the proximity (Area Susceptibility) as a continuous variable and a factor variable indicating whether the entire farm is depopulated within the first month (Quick Farm Culling), whether the first cage is depopulated within the first month (Quick Cage Culling), and whether the depopulation was delayed as baseline (intercept). The baseline model always uses the “slow culling” coefficient as the intercept and the “Area susceptibility (log)” coefficient as the slope, while the other parameters represent the difference from the baseline model coefficients. Note that “Quick Farm Culling” and “Quick Cage Culling” are combined to one factor level in alternative model 2. The marginal effects from the models are shown in Figure 2*.

A model that includes one three-category variable and one continuous variable represents three linear functions *a*+*bx*, where *a* is the intercept and *b* is the slope (on the log scale in this case, because the Poisson part of the model uses a log link function). The coefficient table has a baseline function that uses the (*intercept*) coefficient as the intercept (representing *slow* *culling* in this case) and the coefficient of the continuous variable as the slope (*Area* *susceptibility*). The intercept in the baseline model represents the first level of the factor variable, which here is *slow* *culling*. The rest of the coefficients represent the differences from the baseline model. Hence, the intercept for quick cage culling is the (*intercept*) coefficient + the *Quick* *cage* coefficient, and the slope is the *Area* *susceptibility* (*log*) coefficient + the *Quick* *Cage* *Culling*: *Area* *susceptibility* (*log*) coefficient. The linear function for quick cage culling in the top-ranked model in plain numbers then becomes:

(8) 0.75 + (−1.21) + (1.84+(−0.92) * log(Area  susceptibility))

which can be simplified to:

(9) −0.46+0.92X*log(Area susceptibility)

Here, both the response and the area susceptibility are log-transformed [log(Area susceptibility + 1), for the predictor to avoid negative infinity], and the back-transformed function for the number of new outbreaks ŷ (corresponding to the green line in Figure 2A) becomes:

(10) $$\hat{y}=e^{0.46+0.92*log(AreaSusceptibility+1)}$$

The top-ranked model explained the number of secondary outbreaks as an effect of the three-category variable “culling” (“quick farm culling,” “quick cage culling,” or “slow culling”), a positive effect of area susceptibility, and the interaction between the two. This means that the linear effect of “area susceptibility” for the three different levels of the variable culling had both different slopes and intercepts, although with largely overlapping confidence bands. The effect of area susceptibility was strongest with slow culling and weakest when the entire farm was culled within the first month. The intercept value, however, is slightly higher for quick farm culling than for quick cage culling. This probably stems from the two cases with low area susceptibility, quick farm culling, and three secondary outbreaks (Figure 2A).

Alternative model 1 includes the three-category variable culling and area susceptibility without interaction, only allowing for different intercept values for the factor levels. The results are counter-intuitive, with higher estimates for the category “Quick farm culling” than for “Quick cage culling” and largely overlapping confidence bands. As for the top-ranked model, this counter-intuitive result is probably caused by the two cases with low area susceptibility, quick farm culling, and three secondary outbreaks (Figure 2B).

In alternative model 2, we use whether the first cage was empty after the first month as an explanatory variable that combines the categories “Quick farm culling” and “Quick cage culling” as a binary variable (Quick culling) that represents whether they culled the first cage within the first month or not. The model includes this two-category variable, area susceptibility, and the interaction between the two. The model shows clear differences in how likely the outbreak is to spread to surrounding salmon sea farms if culling is delayed (Figure 2C).

The effect of culling of the entire farm in the top-ranked model and alternative model 1, however, is ambiguous and seems to change between the models, rendering alternative model 2 the most plausible.

## 4. Discussion

We here show that the number of new salmon sea farms infected in outbreaks of ISA correlates positively with contact with other salmon sea farms and that this effect increases with an increase in the time elapsed from suspicion of ISA until the first cage is culled. We argue that the duration of time between suspicion of disease outbreak and the culling of the first cage is a strong indicator of whether the affected farm aimed to depopulate all diagnosed fish with as little delay as possible. Moreover, the significant positive signal in time until culling of the first cage supports the assumption that farmers depopulate the infected cages on the farm site first. From this, we conclude that the time elapsed until culling of the first cage is a good proxy for the time elapsed until culling of infected cages. The results presented herein therefore highlight the fact that infections in fish in aquaculture are at increasing risk of spreading to new salmon sea farms with increasing duration of the infection and that *hypothesis 1* is true: the risk of secondary cases will increase with the time period that a salmon sea farm holds fish with diagnosed ISA. The results further suggest that in order to limit the probability of further spread of the infection, one should prioritize depopulating all the infected cages or cages with high mortality as soon as possible. The area susceptibility is a parameter that also includes the number of fish in the infected farm. We therefore suggest that depopulation of the infected cage with the highest number of fish should be prioritized.

The ambiguous results of farm culling between the top-ranked model and alternative model 1 render any effect of farm culling inconclusive. It is important to note that our data set is limited, with only 31 of the outbreaks spreading to new salmon sea farms, and data from aquaculture contain much variation and noise due to human activity and regulations. Some of these secondary cases may already have been infected when the source outbreak was detected, thus causing the secondary outbreaks affected by culling practices to be even lower in number. The limited amount of data therefore has little power to separate the farm and cage effects. Our results still highlight the importance of prompt action when facing an ISA outbreak and that delaying actions may lead to new outbreaks. We do not seem to have enough data, however, to confirm *hypothesis 2*: culling of the entire salmon sea farm is a better precautionary measure than only culling cages with high mortality or confirmed diagnosis.

According to Aldrin et al. (12), the most important predictor of further spread of infection when genetic similarities between virus strains are left out of the equation is the seaway distance between salmon sea farms. This relationship is assumed to be exponential, and cages within the same salmon sea farm are usually very close to each other. All cages without infected fish in an infected farm are therefore at exceptionally high risk of contracting the infection when compared to other salmon sea farms. The power of our analyses could not establish whether the entire salmon sea farm is the functional epidemiologic unit of an ISA outbreak, but the model with the best fit includes a parameter that emphasizes the effect of seaway distances. Our results therefore support the findings in Aldrin et al. (12) and consequently indicate that seemingly uninfected cages in an infected salmon sea farm might be infected at an early stage when it is difficult to detect the virus. Hence, the best precautionary action against further spread may still be the culling of entire salmon sea farms.

We do not consider economic cost/effect relationships or animal welfare issues herein, and detailed management advice that applies to every outbreak is therefore outside the scope of this paper. We do suggest, however, that cases where only partial culling, e.g., depopulation of only infected or high-mortality cages is chosen, should be followed by intensive monitoring of the remaining fish. We also suggest that the detection probabilities and sensitivity of the methods used in early stages of an infection should be investigated in future research to ensure reasonable monitoring regimes in partially culled salmon sea farms.

## Author Contributions

LQ has been involved with study design, data analysis, statistics, and prepared the manuscript. AK and TL has been involved with data analysis, statistics, manuscript review, and editing. AL had the idea, and has reviewed and edited the manuscript.

### Conflict of Interest

The authors declare that the research was conducted in the absence of any commercial or financial relationships that could be construed as a potential conflict of interest.
